# Prefrontal tDCS Attenuates Self-Referential Attentional Deployment: A Mechanism Underlying Adaptive Emotional Reactivity to Social-Evaluative Threat

**DOI:** 10.3389/fnhum.2021.700557

**Published:** 2021-08-17

**Authors:** Jens Allaert, Maide Erdogan, Alvaro Sanchez-Lopez, Chris Baeken, Rudi De Raedt, Marie-Anne Vanderhasselt

**Affiliations:** ^1^Ghent Experimental Psychiatry Lab, Department of Head and Skin, Ghent University, University Hospital Ghent (UZ Ghent), Ghent, Belgium; ^2^Psychopathology and Affective Neuroscience Laboratory, Department of Experimental Clinical and Health Psychology, Ghent University, Ghent, Belgium; ^3^Research in Developmental Disorders Lab, Department of Experimental Clinical and Health Psychology, Ghent University, Ghent, Belgium; ^4^Department of Clinical Psychology, Universidad Complutense de Madrid, Madrid, Spain; ^5^Department of Psychiatry, Vrije Universiteit Brussel (VUB), Universitair Ziekenhuis Brussel (UZ Brussel), Brussels, Belgium; ^6^Department of Electrical Engineering, Eindhoven University of Technology, Eindhoven, Netherlands

**Keywords:** tDCS, self-referential processing, attentional deployment, emotional reactivity, social-evaluative threat, DLPFC

## Abstract

Social-evaluative threat (SET) – a situation in which one could be negatively evaluated by others – elicits profound (psycho)physiological reactivity which, if chronically present and not adaptively regulated, has deleterious effects on mental and physical health. Decreased self-awareness and increased other-awareness are understood to be an adaptive response to SET. Attentional deployment – the process of selectively attending to certain aspects of emotional stimuli to modulate emotional reactivity – is supported by fronto-parietal and fronto-limbic networks, with the dorsolateral prefrontal cortex being a central hub. The primary aim of the current study was to investigate the effects of active (versus sham) prefrontal transcranial direct current stimulation (tDCS) on self and other-attentional deployment during the exposure to a SET context. Seventy-four female participants received active or sham tDCS and were subsequently exposed to a rigged social feedback paradigm. In this paradigm a series of social evaluations were presented together with a photograph of the supposed evaluator and a self- photograph of the participant, while gaze behavior (time to first fixation, total fixation time) and skin conductance responses (SCRs; a marker of emotional reactivity) were measured. For half of the evaluations, participants could anticipate the valence (negative or positive) of the evaluation *a priori*. Analyses showed that participants receiving active tDCS were (a) slower to fixate on their self-photograph, (b) spent less time fixating on their self-photograph, and (c) spent more time fixating on the evaluator photograph. During unanticipated evaluations, active tDCS was associated with less time spent fixating on the evaluation. Furthermore, among those receiving active tDCS, SCRs were attenuated as a function of slower times to fixate on the self-photograph. Taken together, these results suggest that in a context of SET, prefrontal tDCS decreases self-attention while increasing other-attention, and that attenuated self-referential attention specifically may be a neurocognitive mechanism through which tDCS reduces emotional reactivity. Moreover, the results suggest that tDCS reduces vigilance toward stimuli that possibly convey threatening information, corroborating past research in this area.

## Introduction

Social interactions make up a substantial part of our lives and the need to belong is a fundamental drive in human behavior (Baumeister and Leary, 1995). Situations in which aspects of one’s self could be negatively evaluated by others (social-evaluative threat; SET) elicit profound physiological and psychological reactivity that may – if not adaptively regulated – lead to the development of depression and increase the risk of cardiovascular diseases (Merz et al., 2002; Slavich et al., 2009, 2010; Muscatell et al., 2015). In terms of adaptive reactivity to SET, theories on self-regulation suggest that decreased self-awareness (e.g., attention toward aspects of the self) serves as a self-protective defensive mechanism to attenuate self-referent rejection-related distress (Twenge et al., 2003; Durlik and Tsakiris, 2015). Concurrently, increased other-awareness (e.g., attention toward aspects of others) is suggested to serve as a mechanism promoting prosocial behavior by tuning attention toward the other(s) and taking their perspective, with the aim to repair and maintain social relationships (Hess and Pickett, 2010; Knowles, 2014). Given the implications of SET reactivity on both mental and physical health, it is important to further investigate the proposed neurocognitive mechanisms underlying adaptive SET reactivity, and how these adaptive processes can be promoted.

The flexible regulation of attention toward relevant and irrelevant information both externally and internally (i.e., selective attention and working memory), is of crucial importance in adaptive behavior and mental health (Gross and Thompson, 2007; Chun et al., 2011; Aldao et al., 2015). An important mechanism underlying depression is assumed to be the presence of attentional biases toward negative self-referent information and difficulties to shift attention away from this information, thereby contributing to sustained negative self-referential processing and perpetuating depressive mood (Koster et al., 2011; Joormann and Vanderlind, 2014). Consistent with this view, studies have shown that the ability to disengage attention away from negative information prospectively predicts the onset of depressive symptoms via repetitive negative self-referential thinking (Sanchez-Lopez et al., 2019c; Yaroslavsky et al., 2019). Theories of emotion regulation postulate that attentional deployment is an emotion regulatory process that involves shifting attentional focus toward or away from particular aspects of emotional stimuli, thereby modulating emotional reactivity (Gross and Thompson, 2007). Research employing concurrent fMRI and eye-tracking showed that deploying attention to non-arousing (compared to arousing) aspects of emotional stimuli involves the recruitment of fronto-parietal brain networks and reduces amygdala activity and self-reported negative affect (Ferri et al., 2013, 2016). The prefrontal cortex is essential for executive functioning (e.g., selective attention, working memory, decision making, etc.) and consists mainly of the dorsolateral prefrontal cortex (DLPFC), the medial prefrontal cortex (mPFC) and the orbitofrontal cortex (OFC), with the latter two regions often considered as a relatively uniform structure; the ventromedial prefrontal cortex (vmPFC; Kolb and Whishaw, 2009). The vmPFC and DLPFC are thought to be both involved in emotional processing, with the vmPFC being involved in the generation of emotional reactivity (i.e., emotional arousal) and the DLPFC being more involved in the attribution of positive or negative valence (Nejati et al., 2021). In addition, the vmPFC has been shown to play a central role in the processing of self-referent information (Northoff et al., 2006). Furthermore, the DLPFC plays a role in the interplay between anticipatory and “online” regulatory processes (Seo et al., 2014; De Raedt and Hooley, 2016), as prior expectations surrounding an upcoming emotional stimulus can greatly influence self-regulatory processes when subsequently confronted with that stimulus (Gunther Moor et al., 2010; van der Veen et al., 2019). Taken together, the prefrontal cortex plays an essential role in a wide range of psychological processes related to cognitive flexibility, emotional processing and emotion regulatory processes (Ochsner and Gross, 2005; Kim et al., 2011; Stuss, 2011).

Studies have shown that the causal modulation of the prefrontal cortex and its associated neural networks, via transcranial direct current stimulation (tDCS), can facilitate processes involved in cognitive flexibility (e.g., selective attention, working memory), modulate the interplay between anticipatory and online self-regulatory processes, and produces a wide array of adaptive cognitive and psychological effects in both depressed and healthy populations (Mondino et al., 2015; Dedoncker et al., 2016; Allaert et al., 2020; Smits et al., 2020). TDCS is a low cost and easy to use form of non-invasive brain stimulation that operates through the delivery of a weak electrical current to the scalp, which modulates the membrane potentials of the underlying neurons and thereby influences cortical excitability (Stagg and Nitsche, 2011). For example, it has been shown that left prefrontal tDCS can transiently (a) reduce attentional biases to threat (Ironside et al., 2016), (b) improve attentional disengagement abilities from emotional information (Sanchez-Lopez et al., 2018), and (c) attenuate emotional reactivity to SET (Allaert et al., 2020). However, ecologically valid research that investigates the effects of prefrontal tDCS on the interplay between selective attention processes toward self-referential stimuli and emotional reactivity, and how prior expectations may influence this, is non-existent. One eye-tracking study showed that anodal (vs. sham) left prefrontal tDCS was associated with a transient diminished attentional bias (i.e., less gaze fixation) toward negative emotion-eliciting images, and that this was associated with reduced self-reported state anxiety (Chen et al., 2017). Thus, in view of the importance of impairments in attentional processes toward self-referential information underlying depression, and the existing research gap, the goal of the present study was to investigate the effects of prefrontal tDCS on self-referential attentional processes, their interplay with emotional reactivity, and how prior expectations may influence these.

To address these research objectives, an experimental paradigm featuring SET was employed with the aim to simulate the nature of real-life situations (e.g., social interactions). In this paradigm participants are systematically exposed to self-relevant information (e.g., positive and negative social evaluations), evoking strong psychological and physiological responses that are relevant to the pathogenesis of depression (Slavich et al., 2009, 2010; Vanderhasselt et al., 2018). Participants are led to believe that, based on their self-photograph, strangers have formed first impressions about them, and that they are presented with these explicit social evaluations. During each exposure to an evaluation, a photograph of the participant and of the supposed evaluator is shown, along with the explicit positive or negative evaluation, while participants’ gaze behavior (time to first fixation and total fixation time) to these is measured. Concurrently, emotional reactivity is assessed via the measurement of autonomic changes in skin conductivity (skin conductance responses; SCRs), as SCRs are mediated by amygdala activity and index emotional arousal (Wood et al., 2014; Christopoulos et al., 2019; Allaert et al., 2020). First, based on the self-regulation theories proposing adaptive SET reactivity consists of both decreased self-attention and increased other-attention (Hess and Pickett, 2010; Durlik and Tsakiris, 2015), and given the wide array of research showing adaptive left prefrontal tDCS effects on attention processes and emotional reactivity (Mondino et al., 2015; Smits et al., 2020), we expected that active (in contrast to sham) left prefrontal tDCS would be associated with attenuated self-attention (i.e., slower first fixation to and less total fixation time on this information) and increased other-attention (i.e., faster first fixation to and more total fixation time on this information). We had no clear expectations whether these effects would be modulated by valence, as previous prefrontal tDCS research has shown both valence-independent (Sanchez-Lopez et al., 2018; Allaert et al., 2020), and valence-dependent tDCS effects (Mondino et al., 2015; Nejati et al., 2021). Second, based on the proposed association between prefrontal-mediated attentional processes and emotional reactivity, we expected the attentional processes that are modulated by tDCS to be more strongly associated with attenuated emotional reactivity (assessed via SCRs) among participants receiving active (versus sham) tDCS. Third, in view of the clinical relevance of the interplay between anticipatory processes and “online” self-regulatory processes, and the role of the DLPFC herein, the last objective was to explore whether tDCS differentially affected gaze behavior during expected vs. unexpected social evaluations.

## Materials and Methods

### Participants

Seventy-four healthy female individuals (M_*age*_ = 20.80, SD_*age*_ = 2.11) participated in the study. Given the sex differences in emotional processing and reactivity to social evaluations (McRae et al., 2008; Lithari et al., 2010; Vanderhasselt et al., 2018), only female participants were included, to reduce the sample variability. The sample size was determined based on an *a priori* power analysis. In lack of clear consensual guidelines for power analyses for the employed statistical method (generalized linear mixed models; see data analytic plan), a power analysis was conducted for linear models instead, using the G^∗^Power software (Faul et al., 2007). This estimated a total sample size of *N* = 74 to detect with 80% power the hypothesized between-within interaction effect of a small to moderate magnitude (Cohen’s f = 0.15) using a mixed ANOVA *F*-test. Participants were recruited in the context of a larger research project investigating the effects of left prefrontal tDCS on the interplay between anticipatory and online emotion regulatory processes to social evaluations (Allaert et al., 2020). They were recruited from the general community via internet postings on social media and posters in public places. Selection criteria were (a) right-handed, (b) normal or corrected to normal vision, (c) no current psychiatric or neurological disorders, (d) no current use of psychiatric drugs, (e) no personal or family history of epilepsy, (f) no recent neurosurgery, (g) not pregnant, and (h) no metal or magnetic objects in or around the scalp. Participants were asked not to smoke or ingest caffeine and/or alcohol 2 h prior to the experiment. The study was conducted with the approval of Ghent University’s Medical Ethical Committee and in accordance with the Declaration of Helsinki. Participants provided informed consent at the start of the experiment and received € 15 for participating.

### Materials

#### Social Feedback Paradigm

**FIGURE 1:**
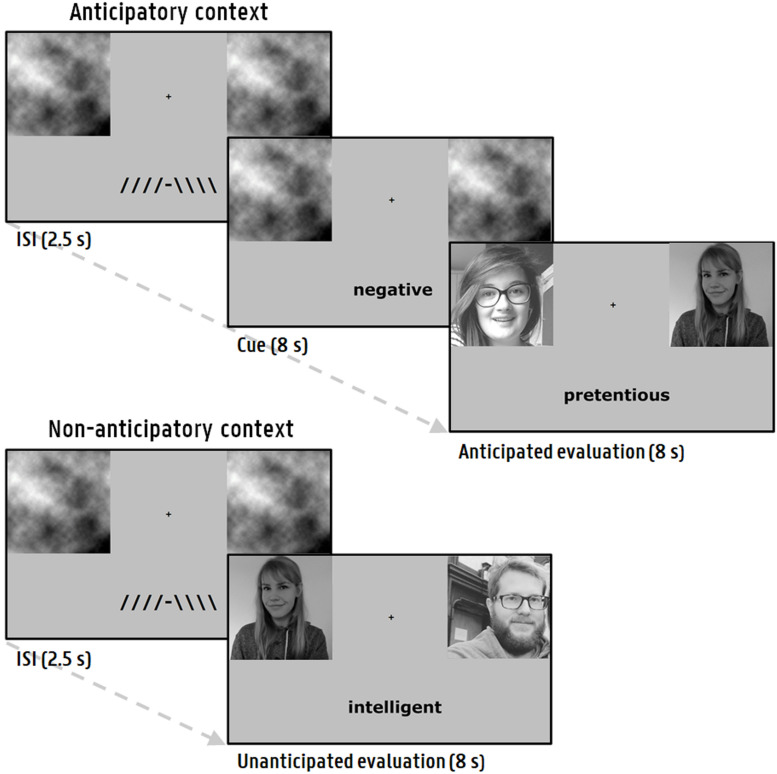
Social feedback paradigm. Every trial started with an ISI and was followed either by an anticipation phase succeeded by an evaluation (anticipatory context), or a direct presentation of an evaluation (non-anticipatory context). During the presentation of an evaluation, a photograph of the participant next to the supposed evaluator was shown.

A paradigm was used in which participants are repeatedly confronted with (experimentally rigged) positive and negative social evaluations (Allaert et al., 2020). Participants were led to believe that the study was part of a larger research project in which research groups from different universities collaborate to investigate how people form first impressions. The participants were briefed that they would have to evaluate participants that were recruited by these other research groups, based on the first impression they form when viewing their photograph. In turn, the participants had to provide a self-photograph so that the supposed participants from the other research groups could evaluate them. Before the paradigm, participants were told that the evaluations of these others will be shown to them. During each social evaluation presentation (8000 ms), participants were shown their self-photograph, the photograph of the supposed evaluator and a negative or positive word (e.g., stupid, attractive). The paradigm consisted of 80 trials (i.e., evaluations), divided in 4 blocks of 20 trials with breaks in between. Each trial started with an inter-stimulus interval (ISI; 2500 ms) displaying a fixation cross. In half of the trials (anticipatory context), participants could correctly anticipate the valence of the social evaluation, prior to its presentation, by means of a cue (8000 ms). In the other half (non-anticipatory context), the evaluation is directly presented after the ISI. Figure 1 shows a visual representation of the experimental sequence for each context. The employed stimuli consisted of 80 unique words (matched on arousal between positive and negative valence) from a validated normative database (see Supplementary materials; Moors et al., 2013) and 80 photographs obtained from volunteers (aged 18 – 30) outside the participant pool. To prevent luminance-evoked pupillary responses, all images were gray-scaled and matched on luminance values via the Matlab SHINE toolbox (Willenbockel et al., 2010). During the ISI, gray-scaled placeholder images with the same luminance values were presented on the location where the subsequent photographs appear (Allaert et al., 2020). The order of the specific combinations of trial features (context, evaluator, gender of evaluator, word, location of the evaluator photograph) was pseudorandom (see Supplementary Materials). During block 1 to 3, the valence of the evaluations was equally distributed, whereas in the last block^1^ negative feedback was more prevalent (80%). The paradigm was programmed in E-prime 2.0 Professional (Psychology Software Tools, Pittsburgh, PA, United States).

#### Transcranial Direct Current Stimulation (tDCS)

**FIGURE 2:**
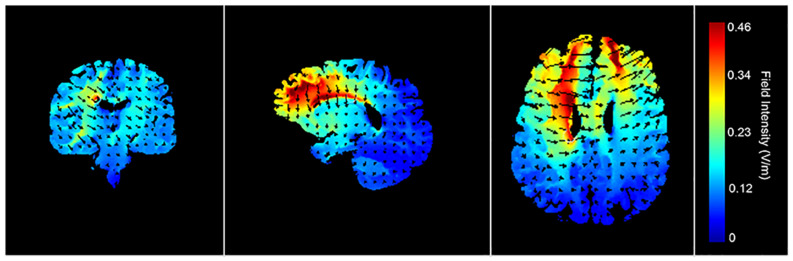
Electric field simulations of the tDCS montage. The electric field simulations show the largest electric field potentials within prefrontal regions.

Transcranial direct current stimulation was applied with a pair of rubber surface electrodes (5 × 7 cm = 35 cm^2^) covered with electrode gel and delivered with a battery-driven stimulator (DC-Stimulator Plus, neuroConn GmbH). The anodal electrode was vertically positioned over F3 (corresponding to the left DLPFC) according to the 10–20 international EEG system, whereas the cathode was placed over the contralateral supra-orbital area (Fp2). This electrode positioning is in accordance with previous tDCS studies on emotional processing and the level B recommendation for treating major depressive disorders (Nitsche et al., 2008; Dedoncker et al., 2016; Lefaucheur et al., 2017). A current of 2 mA (current density = 0.06), with 30 s of ramp up was applied for 20 min (with ramp down at the end). Half of the participants received active tDCS, whereas the other half received sham tDCS (between-subject design). For sham tDCS (i.e., placebo) the current was directly ramped down after the initial ramp up phase (Nitsche et al., 2008). Figure 2 shows a visualization of the electric field simulation of the utilized tDCS montage (Kempe et al., 2014), using Soterix HD-Explore software. The tDCS procedure followed a single-blind design. All involved authors have extensive expertise in the application of tDCS in combination with eye-tracking (Allaert et al., 2019, 2020, 2021).

#### Eye Tracking

Gaze behavior was recorded at a sample rate of 300 Hz with the Tobii TX300 eye tracker (Tobii AB, Stockholm, Sweden) in conjunction with the E-Prime Extensions for Tobii (Psychology Software Tools, Pittsburgh, PA, United States). A standard 9-point calibration sequence was used to calibrate participants’ eye tracking. Fixations were defined as to be at least 100 ms of duration. Time to first fixation (i.e., time elapsed prior to making first fixation on a given AOI, in seconds) and total fixation time (i.e., total time spent fixating on a given AOI, in seconds) were computed for every AOI (self, evaluator, feedback) on every trial, using the PhysioData Toolbox 0.5 (Sjak-Shie, 2018), and served as the primary dependent variables. The first author and experiment leader received training in eye-tracking methodology from the eye tracker manufacturer (Tobii AB, Stockholm, Sweden).

#### Skin Conductance Responses (SCRs)

Electrodermal activity was recorded at a sample rate of 1000 Hz with the Biopac EDA100c amplifier, in conjunction with the Biopac MP150 (Biopac Systems Inc., Santa Barbara, CA, United States). On the amplifier, the gain was set to 5 μS/V, the low pass filter set to 10 Hz, and both high pass filters set to DC mode (off). Two pre-gelled Ag/AgCL electrodes, connected to the amplifier, were placed on the left, non-dominant hand, on the thenar and hypothenar. The data was collected in the Acqknowledge software on an external computer, together with event triggers that were sent by the E-Prime computer to the Biopac STP100c, via a Cedrus StimTracker Duo device (Biopac Systems Inc., Santa Barbara, CA; Cedrus Corporation, San Pedro, CA, United States). Using the Ledalab 3.4.9 MATLAB toolbox (Karenbach, 2005), the data was downsampled to 50 Hz (to increase processing speed), artifact corrected using spline interpolation, smoothed using a 16 sample moving average, and filtered using a first order Butterworth 5 Hz lowpass filter. Continuous decomposition analysis then extracted the phasic information from the skin conductance signals, based on a SCR detection threshold of 0.01 μS (Boucsein et al., 2012), and computed average SCR amplitude (expressed in μS) for every presentation of a social evaluation, which served as the secondary dependent variable.

#### Self-Report Measurements

To ensure comparable active and sham tDCS groups, an online survey assessing various potential confounders was carried out prior to the experiment. These consisted of age, perceived criticism, self-esteem, resilience, the habitual use of various maladaptive and adaptive emotion regulation strategies, symptoms of depression, anxiety and distress, and self-efficacy. These variables could potentially influence reactivity to the social feedback paradigm. Table 1 presents an overview of the descriptive and inferential statistics of these variables. Participants were pseudo-randomly assigned to one of the two groups, in order to have comparable groups based on this survey data. This procedure consisted of a random allocation of participants to groups in the first half of the participant sample. Halfway through data collection the online survey data of planned participants were monitored and based on this data, scheduled participants were allocated to one of the two groups in order to minimize group differences on these variables. Independent *t*-tests showed that the two tDCS groups did not significantly differ on any of these variables (all *p*s > 0.13).

**TABLE 1:** Descriptive statistics and welch two sample *t*-test statistics.

	**Active tDCS**	**Sham tDCS**		
**Variable**	**M (SD)**	**M (SD)**	***t***	***p***
Age	21.16 (2.42)	20.43 (1.69)	1.50	0.14
Perceived criticism PC	6.62 (1.46)	6.27 (1.88)	0.90	0.37
Self-esteem RSES	18.92 (5.52)	19.59 (5.69)	–0.51	0.61
Self-efficacy GSE	28.51 (4.04)	28.76 (5.82)	–0.24	0.81
Resilience CD-RISC	25.54 (5.10)	25.19 (5.82)	0.28	0.78
Cognitive reappraisal ERQ	27.35 (5.89)	28.46 (4.77)	–0.89	0.38
Expressive suppression ERQ	13.30 (4.29)	13.76 (3.83)	–0.49	0.63
Acceptance CERQ	13.81 (2.45)	14.57 (3.45)	–1.09	0.28
Positive reappraisal CERQ	13.65 (3.21)	14.41 (3.66)	–0.94	0.35
Positive refocus CERQ	11.84 (3.02)	11.14 (3.00)	1.00	0.32
Putting into perspective CERQ	14.78 (3.33)	15.08 (2.92)	–0.41	0.68
Refocus on planning CERQ	14.65 (3.43)	15.16 (2.97)	–0.69	0.49
Rumination CERQ	13.68 (4.24)	14.08 (4.05)	–0.42	0.68
Catastrophizing CERQ	7.54 (2.82)	8.46 (2.98)	–1.36	0.18
Self-blame CERQ	13.03 (3.10)	13.16 (2.42)	–0.21	0.83
Other-blame CERQ	8.19 (3.06)	8.54 (3.30)	–0.47	0.64
General distress MASQ30	24.30 (7.91)	25.46 (7.94)	–0.63	0.53
Anhedonic depression MASQ30	25.92 (7.54)	28.92 (9.03)	–1.55	0.13
Anxious arousal MASQ30	17.24 (5.96)	18.11 (6.74)	–0.58	0.56
Rumination RRS	49.27 (12.98)	48.51 (12.88)	0.25	0.80
Brooding RRS	11.27 (3.34)	10.92 (3.21)	0.46	0.65
Reflection RRS	10.84 (3.71)	10.19 (3.64)	0.76	0.45

##### Perceived criticism

Perceived criticism was measured using a 1-item visual analog scale (*“How critical do you think people in your nearest environment – such family, friends, partner – are of you?”*; Masland and Hooley, 2015), ranging from 0 (not at all) to 100 (a lot).

##### Self-esteem

Self-esteem was measured using the Rosenberg Self-Esteem Scale (RSES; Rosenberg, 1965; Franck et al., 2008), a scale consisting of 10 items (e.g., “*I take a positive attitude toward myself*”) rated on a 4-point Likert scale (0 = completely disagree, 3 = completely agree). The scale showed good internal consistency (Cronbach’s α = 0.87).

##### Resilience

Resilience was measured using the Connor-Davidson Resilience Scale (CD-RISC; Connor and Davidson, 2003), a scale consisting of 25 items (e.g., “*I am not easily discouraged by failure*”) rated on a 5-point Likert scale (0 = not true at all, 4 = true nearly all of the time). The scale showed good internal consistency (Cronbach’s α = 0.86).

##### Emotion regulation

The habitual use of maladaptive and adaptive emotion regulation strategies was measured with the Ruminative Response Scale (RRS; Nolen-Hoeksema and Morrow, 1991; Raes et al., 2009), the Emotion Regulation Questionnaire (ERQ; Gross and John, 2003), and the Cognitive Emotion Regulation Questionnaire (CERQ; Garnefski and Kraaij, 2007). The RRS is a 22-item scale rated on a 4-point Likert scale (1 = almost never, 4 almost always), assessing 3 types of responses to depressive feelings; rumination (e.g., *“I think about how sad I feel”*), brooding (e.g., *“Thinking about a recent situation, wishing it had gone better”*), and reflection (e.g., *“Analyze my personality to try to understand why I am depressed”*). The rumination (Cronbach’s α = 0.91) factor showed excellent internal consistency while the brooding (Cronbach’s α = 0.70) and reflection (Cronbach’s α = 0.76) factor showed acceptable internal consistency. The ERQ is a 10-item scale rated on a 7-point Likert scale (1 = completely disagree, 7 = completely agree) and assess the habitual usage of cognitive reappraisal (e.g., *“I control my emotions by changing the way I think about the situation I’m in”*) and expressive suppression (e.g., *“When I am feeling negative emotions, I make sure not to express them”*). The factor expressive suppression (Cronbach’s α = 0.64) showed questionable internal consistency while cognitive reappraisal (Cronbach’s α = 0.76) showed acceptable internal consistency. The CERQ is a 36-item scale rated on a 5-Point Likert scale (1 = almost never, 5 = almost always), assessing 9 types of emotion regulation in response to negative experiences; self-blame (e.g., *“I feel that I am the one to blame for it”*), acceptance (e.g., *“I think that I have to accept the situation”*), rumination (e.g., *“I dwell upon the feelings the situation has evoked in me”*), positive refocus (e.g., *“I think of something nice instead of what has happened”*), refocus on planning (e.g., *“I think of what I can do best”*), positive reappraisal (e.g., *“I think I can learn something from the situation”*), putting into perspective (e.g., *“I think that it all could have been much worse”*), catastrophizing (e.g., *“I often think that what I have experienced is much worse than what others have experienced”*), and other-blame (e.g., *“I feel that others are responsible for what has happened”*). The factors rumination (Cronbach’s α = 0.86), refocus on planning (Cronbach’s α = 0.86), other-blame (Cronbach’s α = 0.87), putting into perspective (Cronbach’s α = 0.81) and positive reappraisal (Cronbach’s α = 0.83) showed good internal consistency, whereas the factors self-blame (Cronbach’s α = 0.71), acceptance (Cronbach’s α = 0.76), positive refocus (Cronbach’s α = 0.76), and catastrophizing (Cronbach’s α = 0.74) showed acceptable internal consistency.

##### Symptoms of depression, anxiety and distress

Symptoms of depression, anxiety, and stress were measured using the short Mood and Anxiety Symptoms Questionnaire (MASQ-30; Wardenaar et al., 2010). This is a 30-item scale rated on a 5-point Likert scale (1 = not at all, 5 = a lot) assessing anhedonic depression (e.g., *“Felt like I had a lot to look forward to”*), anxious arousal (e.g., *“Heart was racing or pounding”*) and general distress (e.g., *“Worried a lot about things”*). The factors anxious arousal (Cronbach’s α = 0.83) and anhedonic depression (Cronbach’s α = 0.84) showed good internal consistency whereas the factor general distress showed acceptable internal consistency (Cronbach’s α = 0.74).

##### Self-efficacy

General self-efficacy was measured with the General Self-Efficacy Scale (GSES; Teeuw et al., 1994), a 10-item scale (e.g., *“I remain calm when facing difficulties because I can rely on my coping abilities”*) rated on a 4-Point Likert scale (1 = completely disagree, 4 = completely agree). The scale showed good internal consistency (Cronbach’s α = 0.82).

##### Mood during the experimental session

To assess changes in mood throughout the experimental protocol, self-reported mood was measured at three time points (pre-stimulation, post-stimulation, and post-task), using six visual analog scales (tiredness, vigorousness, angriness, tension, sadness, and happiness; McCormack et al., 1988) presented on the computer screen, ranging from “totally not” (0) to “very much” (100).

### Protocol

Participants provided informed consent on a webpage and completed the online survey. They were led to believe that they would take part in a study in which the effects of tDCS on the processing of first impressions are investigated. It was stated that they had to first evaluate participants recruited by other collaborating universities, based on the first impression they experience when viewing a photograph of them. In turn, these other students would then evaluate them, based on their self-photograph. On the webpage, participants were presented a series of 20 pictures of strangers along with 4 evaluative descriptive words (2 negative and 2 positive words, obtained from a validated database of Dutch words; Moors et al., 2013). For each picture, participants were asked to indicate which word corresponded the most with the first impression they had formed about the stranger. Afterward, participants could upload a self-photograph. At the end, participants could schedule the experiment. In the laboratory, participants were seated in front of a computer screen and were connected to the physiological recording equipment. Participants underwent active or sham tDCS, and mood states were assessed before and after the stimulation session. Then, the social feedback paradigm started. Finally, mood states were assessed, and participants were debriefed and paid.

### Data Analytic Plan

All data were analyzed in R 3.6.1 (R Core Team, 2013) using (generalized) linear mixed models [(G)LMM]s fitted via the “lmer” and “glmer” functions of the “*lme4*” R package (Bates et al., 2014). These mixed models can handle unbalanced data, account for inter-individual variability in psychophysiological reactivity and offer higher control of type I errors compared to classic (general) linear models (Cnaan et al., 1997; Bagiella et al., 2000; Mallinckrodt et al., 2001; Boisgontier and Cheval, 2016). The implementation of GLMMs requires to specify an underlying distribution of the observed data (e.g., normal, gamma), instead of relying on the assumption that the data follows a normal distribution as in classic linear models (e.g., ANOVA, ANCOVA, repeated measures ANOVA; Jiang and Nguyen, 2021). In addition, when using GLMMs, a link function needs to be specified; this specifies the type of relationship between the independent variables and the dependent variable, such as an identity (linear), logarithmic or inverse relationship. By specifying a link function, data transformations that are often employed in classic linear models are not required. This is advantageous as data transformations alter the original scale of the measurement and may potentially lead to misleading conclusions (Lo and Andrews, 2015). Taken together, GLMMs offer increased flexibility to model data by allowing different types of data distributions and relationships between the predictors and outcome (e.g., linear, logarithmic, and inverse).

**FIGURE 3:**
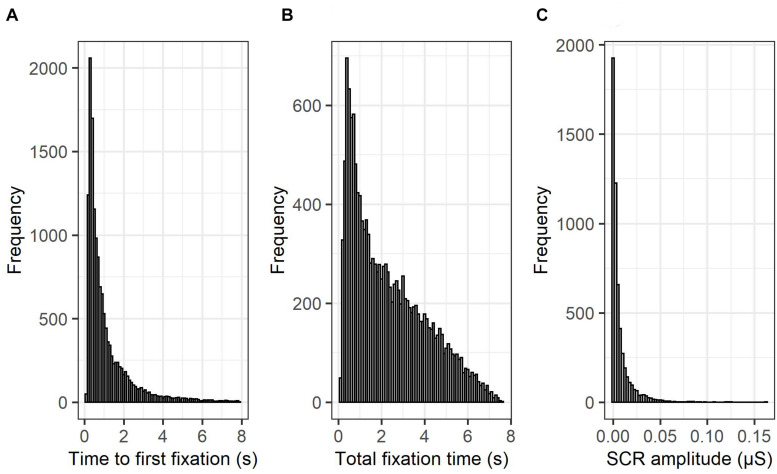
Histograms of time to first fixation, total fixation time, and skin conductance responses. The histograms of the three employed dependent variables [time to first fixation; **(A)**, total fixation time; **(B)**, SCRs, **(C)**] suggest that none follow a normal distribution and that a GLMM approach is warranted.

First, a visual inspection of the histogram of each of the three dependent variables (Time to first fixation; see Figure 3A, Total fixation time; see Figure 3B, SCRs, see Figure 3C) suggested that none of the variables followed a normal distribution, and that a generalized linear mixed modeling approach was warranted. Therefore, to ensure the selection of a distribution and link function that best fits the observed data, a series of (G)LMM models were, based on the Akaike Information Criterion (AIC), compared for each dependent variable (time to first fixation, total fixation time, SCRs; see Table 2), only differing in their specified distribution (normal and gaussian) and link (identity, log and inverse) function. The lowest AIC value indicates the best fitting model of the series. The specific model structure for each of the three dependent variables (time to first fixation, total fixation time, SCRs; see further) was based on our hypotheses and continuous predictors were standardized prior to model fitting. Since a gamma distribution is only compatible with data containing positive numbers (excluding zero), SCR data points containing zero (0.75%) were removed to assess the fit under a gamma distribution. Furthermore, prior to model fitting, gaze data points containing (a) more than 50% missing gaze data, or (b) a time to first fixation to an AOI faster than 100 ms (i.e., anticipatory saccade; Sanchez-Lopez et al., 2019b) were excluded from subsequent analyses (14.75%). In addition, due to technical malfunctions with the SCR recording equipment and the eye-tracker, the SCR data of 5 subjects were corrupted and excluded from further analysis (6.76%). Based on the AIC and consistent with statistical literature, *time to first fixation* (i.e., reaction time data) was best described by a gamma model with a log-link (Lo and Andrews, 2015; Santhanagopalan et al., 2018), whereas *total fixation time* (i.e., duration data) was best described by a gamma model with an identity-link (Hardin and Hilbe, 2007). *SCR amplitude* was best described by a log-gamma model (Braithwaite et al., 2013). The analysis-of-variance tables for these retained models were then computed via the “ANOVA” function of the “*car”* R package, with the sum of squares estimated using the type III approach (Fox et al., 2012). The statistical significance level was set to *p* < 0.05 and *p*-values for the fixed effects were estimated with the “lmerTest” R package, using the Satterthwaite approximations to degrees of freedom (Kuznetsova et al., 2017). For the decomposition of interaction effects, follow-up tests were pairwise comparisons of the estimated marginal means (EMMs) or pairwise comparisons of the EMMs of linear trends (i.e., comparison of slopes), via the “emmeans” and “emtrends” function in the “*emmeans”* R package (Lenth, 2018). To maximize statistical power, follow-up tests for which we had specific directional hypotheses were one-tailed (self and evaluator), whereas the others were two-tailed. Furthermore, *p*-values from the follow-up tests were corrected for multiple comparisons using the false discovery rate correction (Benjamini, 2010).

**TABLE 2:** Model fit (AIC) for dependent variables.

**Dependent variable**	**Normal**	**Log-gamma**	**Identity-gamma**	**Inverse-gamma**
Time to first fixation	40915	22004*	22245	22216
Total fixation time	49468	44054	43598*	44128
SCR amplitude	−23682	−32396*		

*lowest value; lower values indicate better model fit.

To investigate (a) the effects of tDCS on attentional deployment during social evaluations, and (b) whether the possibility of prior anticipation affected these, two GLMMs were fitted with *time to first fixation* (i.e., elapsed time until first fixation on an AOI) and *total fixation time* [i.e., total time spent fixating on an area of interest (AOI)] as respective dependent variables. These GLMMs both featured *group* (active tDCS, sham tDCS), *AOI* (self, evaluator, and feedback), *valence* (negative, positive), *type* (anticipated, non-anticipated) as fixed factors, and *subject* as random intercept.

To investigate how tDCS affects the relationship between tDCS-affected attentional processes and emotional reactivity to social evaluations, one GLMM was fitted with *SCR amplitude* as the dependent variable. This model featured *group* (active tDCS, sham tDCS), *valence* (negative, positive), *type* (anticipated, non-anticipated) as fixed factors, and *subject* as random intercept. Furthermore, based on the results of the previous gaze analyses, the specific observed tDCS effects on the various attentional indices (see results; i.e., *group* × *self-fixation time*, *group* × *evaluator-fixation time*, *group* × *time to first self-fixation*, *group* × *type* × *time to first feedback-fixation*) were entered as predictors in this GLMM.

Finally, to check whether the observed tDCS effects are independent of changes in mood throughout the experimental procedure, six LMMs (for every mood state; tiredness, vigorousness, angriness, tension, sadness, and happiness) were fitted with *group* (active tDCS and sham tDCS), and *time* (pre-stimulation, post-stimulation, and post-task) as fixed factors, and *subject* as random intercept.

Results of the 3 main analyses where tDCS was not implicated are reported in the Supplementary Materials, as these fall outside the scope of the research objectives.

## Results

Participants were blind to their allocation to the tDCS groups as they were unable to correctly guess whether they received active or sham tDCS; the proportion of incorrect guesses (80%) was significantly higher than chance level, (0.50), *p* < 0.001. No adverse effects of tDCS (e.g., headache, fatigue, itching; Matsumoto and Ugawa, 2017) were reported. In addition, there were no significant differences in the time of day of the experimental sessions between the active and sham tDCS groups, *t* = −0.59, *p* = 0.56.

### Effects of tDCS on Time to First Fixation

**FIGURE 4:**
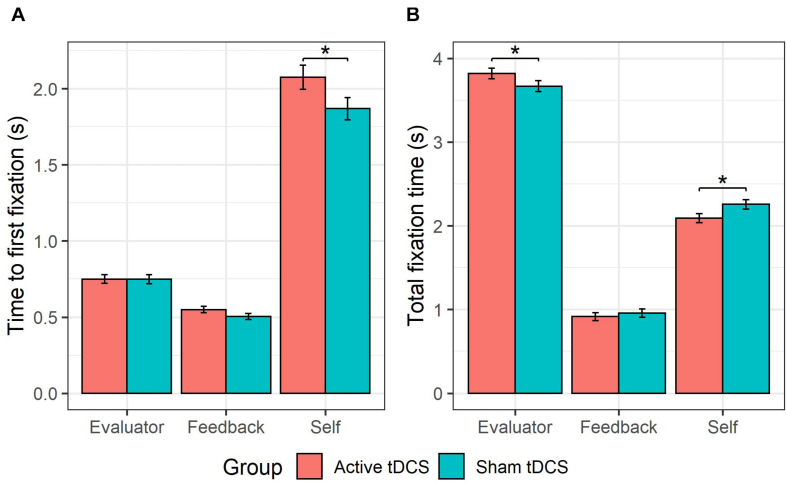
tDCS effects on self- vs. other-attention. The group receiving active (vs. sham) tDCS were slower to fixate on their self-photograph **(A)**, and fixated less on it **(B)**. Furthermore, the active (vs. sham) tDCS group fixated more on the evaluator-photograph **(B)**.

**FIGURE 5:**
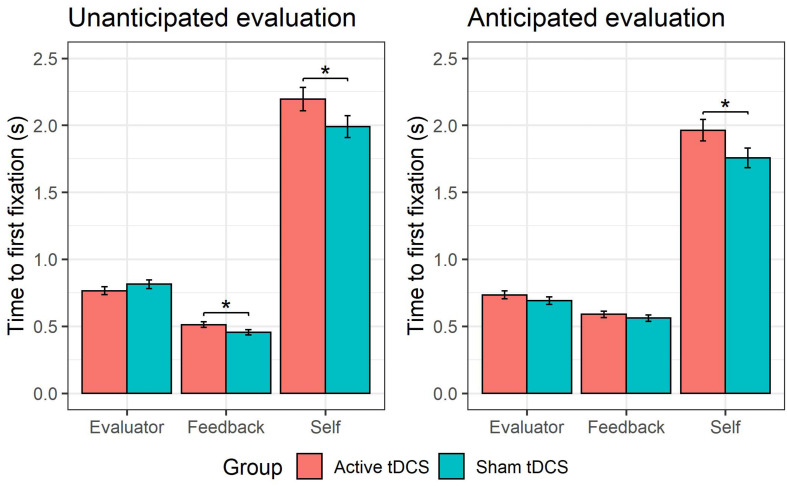
tDCS effect on early attention to unanticipated feedback. The group receiving active (vs. sham) tDCS were slower to fixate on their self-photograph during both anticipated and unanticipated evaluations. During unanticipated evaluations, the group receiving active (vs. sham) tDCS were slower to fixate on the feedback word, whereas this was not the case during anticipated evaluations.

This GLMM showed a *group* × *AOI* interaction (see Figure 4A), χ^2^(2) = 18.54, *p* < 0.001. Follow-up pairwise comparisons probing the effect of *group* for each *AOI* (self, evaluator, feedback) showed that active (versus sham) tDCS was associated with a slower time to fixate on the self-photograph, *b* = 0.10, *SE* = 0.05, *z* = 1.91, *p* = 0.03. There were no significant differences in time to first fixation between active and sham tDCS for the evaluator-photograph, *b* = −0.0003, *SE* = 0.050.05, z = −0.010.01, *p* = 0.500.50, or for the feedback word, b = 0.080.08, SE = 0.06, *z* = 1.52, *p* = 0.13. Furthermore, a higher-order *group* × *AOI* × *type* interaction (see Figure 5) was observed, χ^2^(2) = 13.74, *p* = 0.001. Follow-up pairwise comparisons investigating the effect of *group* on each *AOI* (self, evaluator, feedback) and *type* (anticipated and non-anticipated) showed that active (vs. sham) tDCS was associated with a slower time to fixate on the self-photograph during both the presentation of anticipated, *b* = 0.11, *SE* = 0.06, *z* = 1.91, *p* = 0.03, and non-anticipated evaluations, *b* = 0.10, *SE* = 0.06, *z* = 1.71, *p* = 0.04. This indicates that the tDCS effect on time to fixate on the self-photograph was not modulated by *type*. However, during the presentation of non-anticipated evaluations, active (vs. sham) tDCS was associated with a slower time to fixate on the feedback word, *b* = 0.12, *SE* = 0.06, *z* = 2.07, *p* = 0.04, whereas this was not the case during anticipated evaluations, *b* = 0.05, *SE* = 0.06, *z* = 0.82, *p* = 0.41. This indicates that the tDCS effect on time to fixate on the feedback word was modulated by type. For the evaluator-photograph, no significant differences were present between active and sham tDCS during neither anticipated, *b* = 0.06, *SE* = 0.06, *z* = 1.06, *p* = 0.85, or non-anticipated evaluations, *b* = −0.06, *SE* = 0.06, *z* = −1.08, *p* = 0.14. All remaining tDCS-implicated effects (i.e., *group, group* × *type, group* × *valence, group* × *type* × *valence*, *group* × *valence* × *AOI, group* × *type* × *valence* × *AOI*) were non-significant (all *p*s > 0.06).

### Effects of tDCS on Total Fixation Time

This GLMM showed a *group* × *AOI* interaction (see Figure 4B), χ^2^(2) = 19.98, *p* < 0.001. Follow-up pairwise comparisons probing the effect of *group* for each *AOI* (self, evaluator, feedback) showed that active (versus sham) tDCS was associated with a shorter total fixation time on the self-photograph, *b* = −0.17, *SE* = 0.08, *z* = −2.15, *p* = 0.02, and a longer total fixation time on the evaluator-photograph, *b* = 0.15, *SE* = 0.09, *z* = 1.65, *p* = 0.04. Active (versus sham) tDCS was not associated with a significant difference on total fixation time to the feedback word, *b* = −0.04, *SE* = 0.070.07, *z* = −0.58, *p* = 0.56. All remaining tDCS-implicated effects (i.e., *group, group* × *type, group* × *valence, group* × *type* × *valence, group* × *type* × *AOI*, *group* × *valence* × *AOI, group* × *type* × *valence* × *AOI*) were non-significant (all *p*s > 0.17).

### Effects of tDCS on the Relationship Between Attentional Indices and Emotional Reactivity

**FIGURE 6:**
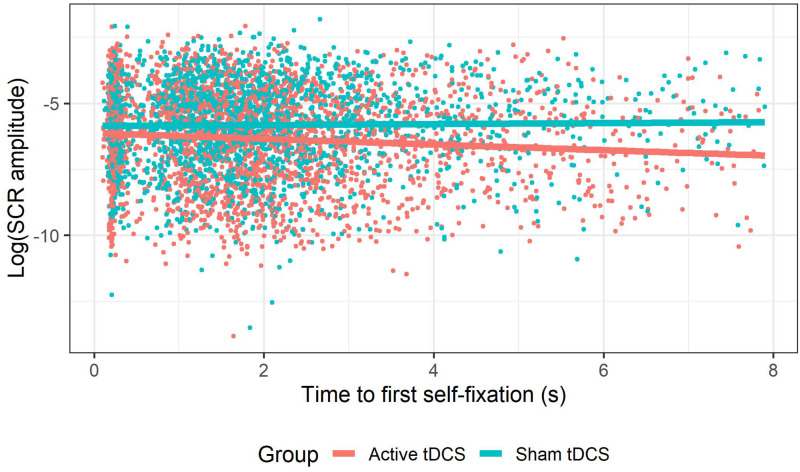
tDCS effect on the relationship between time to first self-fixation and emotional reactivity. Within the group receiving active tDCS, slower times to fixate on the self-photograph were associated with smaller SCR amplitudes, whereas this was not the case among the group receiving sham tDCS.

This GLMM showed a *group* × *time to first self-fixation* interaction (see Figure 6), χ^2^(1) = 14.12, *p* < 0.001. Follow-up tests showed that in the active tDCS group, *time to first self-fixation* was negatively associated with SCR amplitude to social evaluations, *b* = −0.19, *SE* = 0.03, *z* = −6.62, *p* < 0.001, whereas this association was non-significant in the sham tDCS group, *b* = −0.04, *SE* = 0.030.03, *z* = −1.34, *p* = 0.18. Thus, during active (compared to sham) tDCS slower times to self-fixate were associated with attenuated SCRs. All remaining tDCS effects where attentional deployment indices were implicated (*group* × *self-fixation time*, *group* × *evaluator-fixation time*, *group* × *time to first feedback-fixation*, *group* × *type* × *time to first feedback-fixation*) were non-significant (all *p*s > 0.24). For an overview of the tDCS effects on SCRs in which attentional deployment was not investigated, see Allaert et al. (2020).

### Effects of tDCS on Mood Throughout the Experimental Procedure

The LMMs on the mood measures (tiredness, vigorousness, angriness, tension, sadness, and happiness) yielded non-significant effects of *group* and *group* × *time* (all *F*s < 0.88, all *p*s > 0.42), indicating that tDCS did not influence reported mood (across time).

## Discussion

The aim of this study was to investigate the effects of prefrontal tDCS on self- versus other-attention (assessed via eye-tracking) during the confrontation with social evaluations, and how the attentional processes affected by tDCS are linked to emotional reactivity (assessed via SCRs). Additionally, it was explored whether the possibility to anticipate the valence of a social evaluation modulated the tDCS effects.

First, the results showed that participants who received active (versus sham) tDCS were a) slower to fixate on their self-photograph, b) spent less time fixating on their self-photograph, and c) spent more time fixating on the photograph of the evaluator. These findings show that in a context of SET, prefrontal tDCS attenuates attentional processes toward self-referential stimuli, while increasing attention toward other-referential stimuli. These results are in line with previous studies showing that prefrontal tDCS can modulate selective attention to (emotional) stimuli (Mondino et al., 2015; Sanchez-Lopez et al., 2018). Second, analyses investigating the relationship between these attentional processes and emotional reactivity showed that among participants receiving active (versus sham tDCS), emotional reactivity decreased in function of slower times to fixate on their self-photograph. This might suggest that delayed attention toward self-referent information may be a neurocognitive mechanism through which prefrontal tDCS adaptively reduces emotional reactivity in situations featuring SET, as was observed in prior studies (Antal et al., 2014; Allaert et al., 2020; Carnevali et al., 2020). Moreover, this would be in line with past research showing that improved cognitive flexibility (e.g., selective attention and working memory) mediates the effects of tDCS on emotional reactivity to negative or stressful stimuli (Chen et al., 2017). Furthermore, It has been suggested that the attenuation of self-referential processes plays an underlying role in the adaptive effects of prefrontal tDCS (Vanderhasselt et al., 2015a; De Raedt et al., 2017; Hoebeke et al., 2021). Taken together, the current results extend previous findings regarding the link between cognitive flexibility and emotional reactivity (Vanderhasselt et al., 2013; Chen et al., 2017), by more directly measuring processes of clinical interest (i.e., attentional processes in the context of SET) in an ecologically valid experimental paradigm featuring natural responding in absence of task instructions.

Regarding the impact of social feedback expectations on tDCS effects, the results showed that during unanticipated evaluations, participants receiving active (versus sham) tDCS were slower to fixate on the feedback word, whereas this was not the case during anticipated evaluations. Generally, participants tended to first fixate on the feedback word when presented with an evaluation, as this conveys the specific content (e.g., intelligent and pretentious) of the social evaluation. During unanticipated evaluations, participants did not know in advance whether the evaluation would be negative or positive, and by looking at the feedback word they could ascertain whether or not the presented evaluation was self-threatening (Sherman and Cohen, 2006). Following this reasoning, the current result may suggest that prefrontal tDCS decreased attentional bias toward potential threatening stimuli. This would be in line with past research (a) implicating the DLPFC in the regulation of attention toward threatful stimuli (Öhman, 2005; Sagliano et al., 2016), and (b) showing prefrontal tDCS effects in attenuating vigilance toward threatening stimuli (Heeren et al., 2015; Ironside et al., 2016, 2017).

Transcranial direct current stimulation is known to modulate large-scale brain networks rather than brain regions in isolation (Peña-Gómez et al., 2012; Stagg et al., 2013). Furthermore, the electric field distribution is influenced by the placement of both the anode (left DLPFC) and cathode (right supra-orbital area) electrode (Bikson et al., 2010) and the strongest electrical field is suggested to be present somewhere in the middle (mPFC) between the anode and cathode (De Witte et al., 2018). Therefore, it is highly likely that multiple brain regions and networks were modulated by tDCS, including the frontoparietal network (FPN; DLPFC, and posterior parietal cortex), which is involved in the flexible regulation of attention toward relevant and irrelevant environmental information and tuning behavior accordingly (Ptak, 2012; Zanto and Gazzaley, 2013), and the default mode network (DMN; mPFC, posterior cingulate cortex, precuneus, and inferior parietal cortex), which is involved in self-referential processing (Raichle et al., 2001; Davey et al., 2016). This would be in line with past research showing that prefrontal tDCS increases connectivity within the FPN while reducing connectivity within the DMN (Keeser et al., 2011; Peña-Gómez et al., 2012). Interestingly, depression is characterized by excessive self-referential processing associated with hyperconnectivity of the DMN, and impaired cognitive flexibility associated with attenuated FPN connectivity (Qin et al., 2014; Li et al., 2018; Schultz et al., 2018). In addition, in view of the observed tDCS-associated attenuation of SCRs linked to attentional processes, prefrontal tDCS could have modulated (a) neural activity of the mPFC, since mPFC activity has been shown to be associated with SCRs (Critchley et al., 2000; Nagai et al., 2004; Zhang et al., 2012), and (b) fronto-limbic networks, as attentional deployment involves the recruitment of the FPN and an attenuation of amygdala activity (Ferri et al., 2016), which is also associated with SCRs (Wood et al., 2014; Christopoulos et al., 2019). However, in order to clearly elucidate the neural circuitry involved in these tDCS effects, the application of neuro-imaging methods such as functional magnetic resonance imaging or high-density electroencephalogram is warranted.

It should be noted that, under normal conditions, the valence (negative, positive) of social evaluations differentially affects self- and other-attentional deployment patterns (Vanderhasselt et al., 2015b, 2018). However, in the current study, tDCS applied to the prefrontal cortex did not reveal a valence specific effect. Past research has shown mixed results regarding the role of valence on tDCS effects, with some studies showing valence-dependent tDCS effects (Mondino et al., 2015; Nejati et al., 2021), and others showing valence-independent tDCS effects (Sanchez-Lopez et al., 2018). Possibly, in the current study, tDCS could have promoted the employment of a general and sustained attentional strategy to a broader context of social evaluative threat, instead of trial-by-trial adjustments based on valence. Such reasoning would be consistent with research showing that neuro-stimulation of the DLPFC contributed to sustained cognitive resource allocation (Pulopulos et al., 2020), and the DLPFC and associated neural networks being implicated in the implementation of proactive regulatory mechanisms (Vanderhasselt et al., 2009).

The results of the current study could be clinically relevant as reactivity to (perceived) social evaluative threat plays a significant role in mental health (Slavich et al., 2009, 2010). Furthermore, impairments in attentional processes have been identified as mechanisms underlying perpetual negative self-referential processing (i.e., rumination) and pose a vulnerability factor for affective disorders (Koster et al., 2011; Sanchez-Lopez et al., 2019c; Yaroslavsky et al., 2019). Given the therapeutic value of prefrontal tDCS, and its effects on attentional processes and emotional reactivity (De Raedt et al., 2015; Mondino et al., 2015; Smits et al., 2020), the current study suggest that attenuated attention toward self-referent information (e.g., a self-photograph) potentially reflects a mechanism of action underlying the adaptive effects of prefrontal tDCS on SET reactivity. There is a growing body of research suggesting that training emotional attentional flexibility can promote adaptive emotion regulatory processes (e.g., cognitive reappraisal; cognitive reframing of a stimuli or situation to change its meaning and emotional valence), while undermining maladaptive regulatory processes (e.g., rumination; Sanchez-Lopez et al., 2016, 2019a,b). Moreover, combining prefrontal tDCS with attention training shows the largest adaptive effects on cognitive reappraisal and rumination, thereby highlighting the importance of multimodal interventions (Sanchez-Lopez et al., 2020). In future studies the effects of prefrontal tDCS combined with attentional training (i.e., focusing less on yourself) on SET reactivity could be investigated. Taken together, in view of the clinical relevance of SET reactivity and the important role of flexible cognition in mental health, the current results suggest that prefrontal tDCS may promote flexible regulatory processes in response to situations featuring SET.

Besides that this study has several strengths, such as the direct assessment of attentional processes through eye-tracking in an ecologically valid paradigm of social evaluation, some limitations must be acknowledged. Past research has shown that (a) the phase of the menstrual cycle (Horvath et al., 2014; Rudroff et al., 2020), and (b) habitual nicotine use (Mclaren et al., 2018), can influence tDCS effects, and this was not explicitly controlled for in the experimental protocol. Given the employed relatively large sample size, it could be argued that these potential effects were balanced out between the two tDCS groups. Furthermore, although participants were instructed to look at the center fixation cross of the screen prior to the presentation of an evaluation, the paradigm was not explicitly programmed to wait for a fixation on this cross before presenting the evaluation. Consequentially, participants could already be looking at the location of any given AOI prior to its presentation, thereby introducing the possibility of confounding anticipatory gaze behavior. To solve this issue, data points indicative of these anticipatory responses were discarded from subsequent analyses, thereby removing a considerable portion of the observed data. Another limitation is that a between-subject design was employed, with no baseline assessment of attentional deployment prior to either active or sham tDCS. However, this design was chosen to prevent habituation and desensitization to the paradigm, which would be present with a pre-post design. To have a comparable participant pool in the active and sham tDCS group, groups were matched on a series of potential confounders. Furthermore, the usage of GLMMs allowed modeling the effects of inter-individual differences via the inclusion of random effects. Finally, the sample only consisted of females, limiting the generalizability of the findings.

In conclusion, this is the first study to investigate the effects of left prefrontal tDCS on self- and other-attentional deployment (via eye-tracking) in a context featuring SET, and how these are linked to autonomic emotional reactivity. The results suggest that prefrontal tDCS promotes adaptive regulatory attentional processes (reduced self-attention and increased other-attention), and that delayed self-attention may be a specific mechanism of action via which tDCS attenuates emotional reactivity to SET.

## Data Availability Statement

The data and analysis script are available online (https://osf.io/3xjcw/).

## Ethics Statement

The studies involving human participants were reviewed and approved by Ghent University Medical Ethical Committee. The patients/participants provided their written informed consent to participate in this study. Written informed consent was obtained from the individual(s) for the publication of any potentially identifiable images or data included in this article.

## Author Contributions

JA designed the experiment with guidance from M-AV and RD, collected the data, performed statistical analyses with support from ME, and wrote the original manuscript with feedback from M-AV, RD, CB, AS-L, and ME. JA and M-AV revised the manuscript with feedback from RD, CB, AS-L, and ME. All authors contributed to the article and approved the submitted version.

## Conflict of Interest

The authors declare that the research was conducted in the absence of any commercial or financial relationships that could be construed as a potential conflict of interest.

## Publisher’s Note

All claims expressed in this article are solely those of the authors and do not necessarily represent those of their affiliated organizations, or those of the publisher, the editors and the reviewers. Any product that may be evaluated in this article, or claim that may be made by its manufacturer, is not guaranteed or endorsed by the publisher.

## Footnotes

1 This was done to investigate emotional recovery from mainly negative social feedback, however, these results fall outside the scope of the research objectives of this manuscript.
