# Supplementary material for: Prefrontal tDCS Attenuates Self-Referential Attentional Deployment: A Mechanism Underlying Adaptive Emotional Reactivity to Social-Evaluative Threat
Source: Front Hum Neurosci. 2021 Aug 17;15:700557. doi: 10.3389/fnhum.2021.700557 (PMC8416079; doi:10.3389/fnhum.2021.700557)
Supplement: Supplementary file 1 [file Data_Sheet_1.docx]

**Prefrontal tDCS attenuates self-referential attentional deployment: a mechanism underlying adaptive emotional reactivity to social-evaluative threat**

**Jens Allaert^1, 2*^, Maide Erdogan^3^, Alvaro Sanchez-Lopez^4^, Chris Baeken^1, 5, 6^, Rudi De Raedt^2^, Marie-Anne Vanderhasselt^1, 2^**

^1^Ghent Experimental Psychiatry Lab, Ghent University, Department of Head and Skin, University Hospital Ghent (UZ Ghent), Ghent, Belgium

^2^Psychopathology and Affective Neuroscience Lab, Ghent University, Department of Experimental Clinical and Health Psychology, Ghent, Belgium

^3^Research in Developmental Disorders Lab, Ghent University, Department of Experimental Clinical and Health Psychology, Ghent, Belgium

^4^E-Motion Lab, Universidad Complutense de Madrid, Department of Clinical Psychology, Madrid, Spain

^5^Vrije Universiteit Brussel (VUB), Department of Psychiatry, University Hospital UZBrussel, Brussels, Belgium

^6^Eindhoven University of Technology, Department of Electrical Engineering, the Netherlands

***Correspondence:**Corresponding Author
jens.allaert@ugent.be

**Supplementary Materials**

**Social feedback paradigm**

The order of the specific trial feature combinations were pseudo-randomized a priori, with the constraints that a) no more than two trials of the same valence can occur consecutively, and b) no more than two trials of the same context-type can occur consecutively. This resulted in a unique (pseudo-randomized) order for half of the participant group, resulting in the same pool of pseudo-randomized order lists between the active and sham tDCS group. The priori pseudo-randomization was carried out with Mix (van Casteren & Davis, 2006) in conjunction with in-house custom MATLAB scripts. Table 1 displays the used Dutch feedback words, along with corresponding English translations and normative valence and arousal ratings (Moors et al., 2013).

| **Table 1. Stimuli normative ratings** | | | |
| --- | --- | --- | --- |
| **Dutch** | **English** | **Arousal** | **Valence** |
| Betrouwbaar | Reliable | 3.91 | 6.22 |
| Intelligent | Intelligent | 4.41 | 6.22 |
| Getalenteerd | Talented | 4.88 | 6.20 |
| Aantrekkelijk | Attractive | 4.86 | 6.19 |
| Oprecht | Sincere | 4.03 | 6.11 |
| Sympathiek | Sympathetic | 4.48 | 6.02 |
| Loyaal | Loyal | 3.95 | 5.92 |
| Aangenaam | Pleasant | 3.72 | 5.94 |
| Creatief | Creative | 4.92 | 5.80 |
| Ruimdenkend | Broad-minded | 4.38 | 5.67 |
| Eerlijk | Honest | 4.05 | 6.16 |
| Levenslustig | Canty | 5.92 | 6.13 |
| Talentvol | Talented | 5.03 | 6.13 |
| Ontspannen | Relaxed | 2.22 | 6.03 |
| Vriendelijk | Friendly | 4.14 | 6.16 |
| Warmhartig | Warm-hearted | 3.97 | 6.16 |
| Sociaal | Social | 4.89 | 5.92 |
| Intellectueel | Intellectual | 4.33 | 5.91 |
| Behulpzaam | Helpful | 4.56 | 5.84 |
| Attent | Thoughtful | 4.39 | 5.80 |
| Competent | Competent | 4.54 | 5.70 |
| Zelfzeker | Confident | 4.56 | 5.67 |
| Assertief | Assertive | 5.41 | 5.55 |
| Verdraagzaam | Tolerant | 3.36 | 5.34 |
| Rechtvaardig | Just | 3.97 | 5.91 |
| Slim | Smart | 4.48 | 5.86 |
| Bescheiden | Modest | 2.84 | 5.05 |
| Beleefd | Polite | 3.59 | 5.72 |
| Sociaal | Social | 4.89 | 5.92 |
| Verantwoordelijk | Responsible | 4.94 | 5.30 |
| Bekwaam | Skilled | 4.36 | 5.67 |
| Goedaardig | Benign | 3.92 | 5.78 |
| Vriendelijk | Friendly | 4.14 | 6.02 |
| Gedisciplineerd | Disciplined | 4.10 | 5.30 |
| Onbekwaam | Incompetent | 3.31 | 2.44 |
| Asociaal | Asocial | 3.78 | 2.11 |
| Hebzuchtig | Greedy | 4.94 | 2.14 |
| Wraakzuchtig | Vindictive | 5.5 | 1.92 |
| Vijandig | Hostile | 5.66 | 1.95 |
| Doelloos | Aimless | 2.766 | 2.44 |
| Bedrieglijk | Deceptive | 4.42 | 2.27 |
| Slordig | Sloppy | 3.75 | 2.72 |
| Overgevoelig | Hypersensitive | 5.17 | 2.88 |
| Neurotisch | Neurotic | 4.97 | 2.20 |
| Onbetrouwbaar | Unreliable | 4.32 | 1.88 |
| Kwetsbaar | Vulnerable | 3.33 | 2.81 |
| Pretentieus | Pretentious | 4.46 | 2.85 |
| Onsympathiek | Unsympathetic | 3.72 | 2.14 |
| Onverdraagzaam | Intolerant | 4.25 | 2.08 |
| Harteloos | Heartless | 3.88 | 1.91 |
| Wanhopig | Desperate | 4.72 | 2.08 |
| Zelfzuchtig | Selfish | 4.27 | 2.33 |
| Onbeleefd | Rude | 4.57 | 2.38 |
| Onredelijk | Unreasonable | 4.23 | 2.42 |
| Wantrouwig | Suspicious | 4.51 | 2.53 |
| Bevooroordeeld | Prejudiced | 4 | 2.45 |
| Preuts | Prudish | 3.02 | 2.92 |
| Haatdragend | Hateful | 1.69 | 5.19 |
| Leugenaar | Liar | 1.78 | 4.67 |
| Ontrouw | Infidelity | 1.80 | 5.00 |
| Boosaardig | Malicious | 1.98 | 5.17 |
| Eenzaam | Lonely | 2.05 | 2.33 |
| Ondankbaar | Ungrateful | 2.06 | 3.66 |
| Jaloers | Jealous | 2.16 | 5.03 |
| Bekrompen | Narrow-minded | 2.25 | 3.48 |
| Arrogant | Arrogant | 2.27 | 5.02 |
| Zelfingenomen | Smug | 2.31 | 4.06 |
| Naïef | Naïve | 2.69 | 3.27 |
| Belabberd | Wretched | 2.36 | 3.13 |
| Oninteressant | Uninteresting | 2.48 | 2.45 |
| Oppervlakkig | Superficial | 2.69 | 2.89 |
| Onintelligent | Untelligent | 2.44 | 3.03 |
| Gewelddadig | Violent | 1.92 | 6.19 |
| Onaangenaam | Unpleasant | 2.14 | 4.25 |
| Verwaand | Conceited | 2.27 | 3.84 |
| Incompetent | Incompetent | 2.44 | 3.31 |
| Sukkelig | Awkward | 2.72 | 3.21 |
| Onoprecht | Insincere | 2.09 | 3.84 |
| Egoïstisch | Selfish | 1.94 | 4.22 |
| Onvolwassen | Immature | 2.69 | 4.11 |

**Non-tDCS implicated results**

**Time to first fixation**

In addition to the tDCS effects reported in the main manuscript, this model also featured a significant effect of *valence*, *χ*^2^(1) = 7.63, *p* = .01, *AOI*, *χ*^2^(2) = 10492.68, *p* < .001, *type* × *AOI, χ*^2^(2) = 152.13, *p* < .001, and *valence* × *AOI, χ*^2^(2) = 27.16, *p* < .001. However, these effects were accounted by a higher order *type* × *valence* × *AOI* interaction, *χ*^2^(2) = 9.69, *p* = .01. Follow-up tests showed that, during both anticipated, *b* = .06, *SE* = .03, *z* = 2.35, *p* = .02, and unanticipated social evaluations, *b* = .09, *SE* = .02, *z* = 3.84, *p* < .001, participants were slower to fixate on the evaluator photograph during negative evaluations, compared to positive evaluations. During anticipated social evaluations, participants were faster to fixate on the feedback when it was negative, compared to positive, *b* = -.11, *SE* = .03, *z* = -3.70, *p* < .001. This was not the case during unanticipated social evaluations, *b* = .007, *SE* = .03, *z* = .27, *p* = .79. Finally, during anticipated social evaluations, participants were slower to fixate on their self-photograph during negative evaluations, compared to positive evaluations, *b* = .09, *SE* = .03, *z* = 3.24, *p* < .01. This was not the case during unanticipated social evaluations, *b* = .03, *SE* = .03, *z* = 1.36, *p* = .17

**Total fixation time**

In addition to the tDCS effects reported in the main manuscript, this model also featured a significant effect of *AOI*, *χ*^2^(2) = 10351.71, *p* < .001, and this effect was accounted by a higher order *type* × *AOI* interaction*, χ*^2^(2) = 14.88, *p* < .001, as well as a *valence* × *AOI* interaction*, χ*^2^(2) = 52.21, *p* < .001. For the *type* × *AOI* interaction, follow-up tests showed that participants spent more time fixating on their self-photograph when the evaluation was anticipated, compared to unanticipated, *b* = .13, *SE* = .04, *z* = 3.51, *p* < .001. This pattern was not present for the evaluator AOI, *b* = -.01, *SE* = .06, *z* = -.09, *p* = .93, or the feedback AOI, *b* = -.02, *SE* = .02, *z* = -1.62, *p* = .11. For the *valence* × *AOI* interaction, follow-up tests showed that participants spent more time fixating on the feedback during negative, compared to positive evaluations, *b* = .11, *SE* = .02, *z* = 7.03, *p* < .001. Participants also spent more time fixating on their self-photograph during negative, compared to positive evaluations, *b* = -.18, *SE* = .04, *z* = -4.91, *p* < .001, whereas there was no difference between negative and positive feedback in total fixation time towards the evaluator, *b* = .05, *SE* = .06, *z* = .87, *p* = .38.

**Relationship between tDCS-affected attentional indices and emotional reactivity**

In addition to the tDCS effects reported in the main manuscript, this model also showed a significant effect of *type*, *χ*^2^(1) = 18.97, *p* < .001. The follow-up test showed that SCRs were higher during unanticipated evaluations, compared to anticipated evaluations, *b* = -.17, *SE* = .04, *z* = -4.36, *p* < .001. In addition, this model showed a significant effect of *self-fixation time*, *χ*^2^(1) = 60.69, *p* < .001, *evaluator-fixation time, χ*^2^(1) = 58.60, *p* < .001, *time to first self-fixation*, *χ*^2^(1) = 31.86, *p* < .001, and *time to first feedback-fixation*, *χ*^2^(1) = 10.12, *p* < .001. A faster *time to first self-fixation* was associated with a larger SCR, β = -.11, *SE* = .02, *t* = -5.64, *p* < .001 and a faster *time to first feedback-fixation* was associated with a smaller SCR, β = .07, *SE* = .02, *t* = 5.64, *p* = .001. Furthermore, longer *evaluator-fixation time,* β = -.22, *SE* = .03, *t* = -7.66, *p* < .001, and longer *self-fixation time*, β = -.21, *SE* = .03, *t* = -7.79, *p* < .001, were associated with a smaller SCR.

**References**

Connor, K. M., & Davidson, J. R. (2003). Development of a new resilience scale: the Connor-Davidson Resilience Scale (CD-RISC). *Depress Anxiety, 18*(2), 76-82. doi:10.1002/da.10113

Garnefski, N., & Kraaij, V. (2007). The cognitive emotion regulation questionnaire. *European Journal of Psychological Assessment, 23*(3), 141-149.

Gross, J. J., & John, O. P. (2003). Individual differences in two emotion regulation processes: implications for affect, relationships, and well-being. *Journal of Personality and Social Psychology, 85*(2), 348.

Masland, S. R., & Hooley, J. M. (2015). Perceived criticism: A research update for clinical practitioners. *Clinical psychology: Science and practice, 22*(3), 211-222.

Moors, A., De Houwer, J., Hermans, D., Wanmaker, S., van Schie, K., Van Harmelen, A. L., . . . Brysbaert, M. (2013). Norms of valence, arousal, dominance, and age of acquisition for 4,300 Dutch words. *Behav Res Methods, 45*(1), 169-177. doi:10.3758/s13428-012-0243-8

Nolen-Hoeksema, S., & Morrow, J. (1991). A prospective study of depression and posttraumatic stress symptoms after a natural disaster: The 1989 Loma Prieta earthquake. *Journal of Personality and Social Psychology, 61*(1), 115-121. doi:<http://dx.doi.org/10.1037/0022-3514.61.1.115>

Rosenberg, M. (1965). *Society and the adolescent self-image*. Princeton, N.J.: Princeton University Press.

Schwarzer, R., & Jerusalem, M. (2010). The general self-efficacy scale (GSE). *Anxiety, Stress, and Coping, 12*(1), 329-345.

van Casteren, M., & Davis, M. H. (2006). Mix, a program for pseudorandomization. *Behavior Research Methods, 38*(4), 584-589.

Wardenaar, K. J., van Veen, T., Giltay, E. J., de Beurs, E., Penninx, B. W., & Zitman, F. G. (2010). Development and validation of a 30-item short adaptation of the Mood and Anxiety Symptoms Questionnaire (MASQ). *Psychiatry Res, 179*(1), 101-106. doi:10.1016/j.psychres.2009.03.005
